# *Neurospora crassa* Protein Arginine Methyl Transferases Are Involved in Growth and Development and Interact with the NDR Kinase COT1

**DOI:** 10.1371/journal.pone.0080756

**Published:** 2013-11-19

**Authors:** Daria Feldman, Carmit Ziv, Rena Gorovits, Michal Efrat, Oded Yarden

**Affiliations:** Department of Plant Pathology and Microbiology, The R.H. Smith Faculty of Agriculture, Food and Environment, The Hebrew University of Jerusalem, Rehovot, Israel; Oregon State University, United States of America

## Abstract

The protein arginine methyltransferaseas (PRMTs) family is conserved from yeast to human, and regulates stability, localization and activity of proteins. We have characterized deletion strains corresponding to genes encoding for PRMT1/3/5 (designated *amt-1*, *amt-3* and *skb-1*, respectively) in *Neurospora crassa*. Deletion of PRMT-encoding genes conferred altered Arg-methylated protein profiles, as determined immunologically. Δ*amt-1* exhibited reduced hyphal elongation rates (70% of wild type) and increased susceptibility to the ergosterol biosynthesis inhibitor voriconazole. In ▵*amt-3*, distances between branches were significantly longer than the wild type, suggesting this gene is required for proper regulation of hyphal branching. Deletion of *skb-1* resulted in hyper conidiation (2-fold of the wild type) and increased tolerance to the chitin synthase inhibitor polyoxin D. Inactivation of two Type I PRMTs (*amt-1* and *amt-3*) conferred changes in both asymmetric as well as symmetric protein methylation profiles, suggesting either common substrates and/or cross-regulation of different PRMTs. The PRMTs in *N. crassa* apparently share cellular pathways which were previously reported to be regulated by the NDR (Nuclear DBF2-related) kinase COT1. Using co-immunprecipitation experiments (with MYC-tagged proteins), we have shown that SKB1 and COT1 physically interacted and the abundance of the 75 kDa MYC::COT1 isoform was increased in a Δ*skb-1* background. On the basis of immunological detection, we propose the possible involvement of PRMTs in Arg-methylation of COT1.

## Introduction

Methylated derivatives of arginine were identified 45 years ago [1]. Since then, protein methylation has been recognized as a post translational modification that plays regulatory roles in signal transduction, nucleic transport, activation and repression of genes and mRNA splicing [2]. Arginine methylation is catalyzed by protein arginine methyltransferases (PRMTs) that since being identified [3], [4] have been found in the genomes of many eukaryotic organisms [5]. The PRMTs are divided into four major types. Type I and II (conserved from yeast to human) are responsible for monomethylation. They catalyze asymmetric (aDMA) and symmetric (sDMA) dimethylarginine, respectively. Type III and IV PRMTs catalyze the monomethylation of arginine residues and the formation of monomethylarginine on guanidinium nitrogen, respectively. So far, Type III and IV PRMTs have only been found in higher eukaryotes [2]. In human cells there are nine genes considered to encode for PRMTs. Among them, only PRMT1 and PRMT3 (Type I), and PRMT5 (Type II) are conserved through eukaryotic evolution, including members of the fungal kingdom [6].

In fungi, PRMTs have been shown to be involved in several cellular functions. For example, PRMT1 has been shown to be important for coping with oxidative stress. In *Aspergillus nidulans*, a concentration of 3 mM H_2_O_2_ resulted in more than 50% growth retardation of Δ*rmtA.* At 6 mM H_2_O_2,_ conidiation was completely abolished [7]. PRMT1 was also shown to be important for hyphal development in *Coprinopsis cinerea* [8] and for pathogenicity in *Fusarium graminearum,* where DON mycotoxin production and virulence on flowering wheat heads were reduced, and increased sensitivity to oxidative and membrane stresses was observed [9]. PRMT5 was found to play a role in cell morphology in *Schizosaccharomyces pombe* [10] and *Saccharomyces cerevisiae* [11], in the increased sensitivity to oxidative stress and elevated temperature in *A. nidulans* [7] and required for full virulence in *F. graminearum* [9]. So far, deletion strains of PRMT3 have not been shown to affect morphology in any of the fungi analyzed [7], [9].

The NDR (Nuclear Dbf2-related) protein kinases are part of the AGC kinase family and belong to the Ser/Thr kinase subgroup [12]. NDR kinases are essential components of important cellular processes such as morphological switches, cell proliferation, mitotic exit and apoptosis in a variety of eukaryotic organisms, and are regulated by co-activators of the MOB (Mps1-one binder) family [13]. *N. crassa* COT1 is the founding member of the NDR kinase family, and a single amino acid substation (within the catalytic domain) results in slow hyphal elongation accompanied by hyperbranching at temperatures above 32°C [14]–[16]. COT1 kinase was shown to be regulated both transcriptionally [17] as well as post transcriptionally, where phosphorylation was shown to be involved in the COT1-dependent regulation of hyphal elongation, branching and of asexual development [15]. In *S. pombe*, the COT1 homologue Orb6 has been shown to physically associate with Skb1, the homologue of the human PRMT5 [18].

In the present study we report on the involvement of PRMTs as mediators of post translational modifications that affect growth and development in *N. crassa* and focus on the possible cross-talk between arginine methylation and phosphorylation, specific to the interactions between PRMTs and the NDR kinase COT1. We characterized all of the three predicted classical PRMT-encoding genes in this fungus and examined their involvement in growth rate, branching, sexual reproduction and cell wall integrity. Our results indicate that COT1 undergoes methylation on arginine residues, and that there is a physical and as well as a genetic interaction between SKB1 and COT1.

## Materials and Methods

### Strains, media and growth conditions

General procedures and media used in the handling of *N. crassa* have been previously described [19] or are available through the Fungal Genetics Stock Center, (www.fgsc.net/Neurospora/NeurosporaProtocolGuide.html). *N. crassa* strains used in this study are listed in Table 1. Strains were grown in either liquid or solid (supplemented with 1.5% agar) Vogel's minimal medium with 1.5% (w/v) sucrose. When required, the medium was supplemented with 100 µg/ml Histidine (Sigma Aldrich), 10 µg/ml hygromycin B (Calbiochem, Riverside, CA) or 10 µg/ml Nourseothricin (Werner BioAgents, Jena, Germany). For antifungal inhibition experiments, the medium was supplemented with either 6.5 mM Polyoxin D (Kaken Chemical Company, Tokyo, Japan), 4.75 µM Caspofungin (Merck Research Laboratories, Rahway), 12.23 mM Voriconazole (Pfizer Inc., Sandwich, England) or NaCl (0.5–1.2 M) (Merck Research Laboratories, Rahway).

**Table 1:** *Neurospora crassa* strains used in this study.

Strain	Genotype	Source
Wild-type	*74-OR23-1 A*	FGSC#987
Wild-type	*ORS-SL6 a*	FGSC#4200
Δ*mus-52;his-3*	Δ*mus-52::barR;his-3*	FGSC#9720
*cot-1 ts*	*cot-1(C102t) A*	FGSC #4065
*cot-1 ts*	*cot-1(C102t) a*	FGSC #4066
MYC::COT1	*myc(start)::cot-1*	[15]
Δ*mob-2a*	Δ*mob-2a hygR*	FGSC #11296
Δ*mob-2b*	Δ*mob-2b hygR*	FGSC #13575
Δ*amt-1*	Δ*amt-1 hygR*	FGSC #11848
Δ*amt-3*	Δ*amt-3 hygR*	FGSC #11832
Δ*skb-1*	Δs*kb-1 hygR*	This study
Δ*amt-1;* Δ*amt-3*	Δ*amt-1;* Δ*amt-3 hygR*	This study
Δ*amt-1;* Δ*skb-1*	Δ*amt-1;* Δ*skb-1 hygR*	This study
Δ*amt-1;cot-1(*MYC::COT1)	*▵amt-1; myc(start)::cot-1 hygR*	This study
Δ*amt-3;cot-1(*MYC::COT1)	Δ*amt-3; myc(start)::cot-1 hygR*	This study
Δ*skb-1;cot-1(*MYC::COT1)	*▵skb-1; myc(start)::cot-1 hygR*	This study
Δ*amt-1;*Δ*amt-3;cot-1(*MYC::COT1)	Δ*amt-1;*Δ*amt-3; myc(start)::cot-1 hygR*	This study
Δ*amt-1;*Δ*skb-1;cot-1(*MYC::COT1)	Δ*amt-1;*Δ*skb-1; myc(start)::cot-1 hygR*	This study
Δ*amt-1;cot-1 ts*	Δ*amt-1; cot-1(C102t) hygR*	This study
Δ*amt-3;cot-1 ts*	Δ*amt-3; cot-1(C102t) hygR*	This study
Δ*skb-1;cot-1 ts*	Δ*skb-1; cot-1(C102t) hygR*	This study
Δ*amt-1; ▵amt-3;cot-1 ts*	Δ*amt-1;* Δ*amt-3; cot-1(C102t) hygR*	This study
Δ*amt-1;* Δ*skb-1;cot-1 ts*	Δ*amt-1;* Δ*skb-1; cot-1(C102t) hygR*	This study
*COT1::GFP*	*cot-1::sgfp+*	This study
Δ*skb-1;COT1::GFP*	Δ*skb-1; cot-1::sgfp+ hygR*	This study
*SKB1::MYC*	*myc::skb-1*	This study
Δ*skb-1; SKB1::MYC*	Δ*skb-1; myc::skb-1 NatR*	This study

Deletion of *skb-1* was performed using the *Neurospora* genome project Gene Knockout Kit (obtained from the FGSC) according to Colot *et al*. [20]. Briefly, the construct for homologous gene replacement of NCU01613.3 was provided by the *Neurospora* knockout project. The cassette was PCR-amplified using the KO-F and KO-R primers and was transformed into the *N. crassa* Δ*mus-52* strain, by electroporation. Transformants were screened for their ability to grow in medium containing hygromycin B, and crossed with a wild-type strain (FGSC#987). Progeny from these crosses were screened for their lack of ability to grow on Basta (to eliminate the Δ*mus-52::barR^+^* background). The deletion mutation was verified by Southern blot and PCR analyses.

A MYC-tagged version of SKB1 was constructed, utilizing the genomic *skb-1* coding sequence along with upstream sequence that contained the promoter. This *skb-1* fragment was obtained by PCR amplification with primers 5'-TCCAAGGAGACGTCAAGACCG and 5'GGTGTAAGTGGGCATCGGAAATAA T. The amplicon was inserted into a pDrive vector (Qiagen, Hilden, Germany) to create pME1. The promoter region of *skb-1* was excised from pME1 by *HincII* and *HindIII* and inserted into pBluescript (Stratagene) to create pME5. The predicted *skb-1* ORF was amplified from pME1 using the primers 5'-AATGAGGGGTCACAGTGATAAGCTT and 5'-CGCGGATCCCGACCTAG CCAGCCTTGC and inserted into pME5 using *Hind*III and *Bam*HI to create pME9. The incorporation of the 6MYC tag resulted in the introduction of an *Nsi*I restriction site at both ends of the product, which was ligated into a pDrive vector to create pME8. The 6MYC tag was inserted at the 5′ region of *skb-1* in pME9, at the *Nsi*I site, to create pME10. The correct orientation and context of the insertion was verified by sequencing. pME10 was linearized by *Bam*HI and co-transformed by electroporation into *N. crassa* along with a 3.3-kb *Xba*I fragment from pMP6, containing a hygromycin-resistance cassette. The colonies were screened for their ability to grow on hygromycin and Southern and western-blot analyses (using anti-Myc antibodies) verified the presence of the MYC-tagged SKB1 with the expected molecular mass. To verify proper activity of the fused SKB1::MYC protein, pME9 and pME10 were co-transformed with pNV15 [21], containing a Nourseothricin resistance cassette for phenotypic complementation of Δ*skb-1*.

For constructing COT1::GFP, a unique *NdeI* site was introduced into pCZ13 [which is the genomic *SmaI/EcoRI cot-1* fragment from pOY18 [16] ligated into pUC118] upstream of the stop codon, by using the primers 5′-CAA CTT CCG ACA TAT GCG TTA C-3′ and 5′-GCA TAT GTC GGA AGT TGT TGT C-3′ to create pCZ13-NdeI. The pCZ13-NdeI, containing the *cot-1* wild-type allele, was capable of complementing the *cot-1 ts* mutant. The sGFP sequence was PCR-amplified from pMF272 [22] using the primers: 5′-GGT TAA TTC ATA TGG TGA GCA AG-3′ and 5-CAT ATG GAA TTC TTA CTT GTA CAG CTC-3′, while introducing an *Nde*I restriction site at both ends of the product, and ligated into a pDrive vector (QIAgen, Hilden, Germany) to create pCZ91. Both pCZ91 and pCZ13-NdeI were digested with *Nde*I and the *gfp* encoding fragment was ligated into pCZ13-NdeI to create pCZ92. Proper integration of the *gfp* tag at the 5′ of *cot-1* CDS was verified by sequencing the resulting plasmid. The resulting pCZ92 plasmid was linearized with *Xho*I and co-transformed into the *cot-1*;Δ*mus-52;his-3* strain along with the *Hind*III*/Bam*HI genomic fragment that can complement *his-3* auxotrophy. Transformants were screened for their ability to grow in medium lacking L-His, at 34°C. The resulting COT1::GFP transformants were screened by PCR to verify homologous recombination. Transformants carrying a single GFP tagged *cot-1* allele at *cot-1* locus were further crossed with *N. crassa cot-1* ts, and screened for their ability to grow at 34°C without L-His and for their sensitivity to Basta (to eliminate the Δ*mus-52::bar^R^* background). Western-blot analysis using rabbit polyclonalanti-GFP antibodies ab290 (Abcam, Cambridge, United Kingdom) verified the presence of GFP-tagged COT1 with the expected molecular mass.

### Determining growth rate, length between branches and conidiation rate

For growth-rate measurements, race tubes containing Vogel's sucrose minimal medium were inoculated with 10 µl of a conidial suspension (2×10^6^ conidia/ml). The race tubes were incubated for several days at 34°C and growth was measured twice daily.

To determine the length between branches, glass slides covered with Vogel's sucrose minimal medium were inoculated with conidia of the different *N*. *crassa* and incubated overnight at 34°C. The edges of the growing colonies were observed microscopically and the entire colony edge was documented in ca. 20 photographs. Each photograph was analyzed using ImageJ 1.37V (Rasband, W.S., U.S. National Institutes of Health, Bethesda, MD, http://rsb.info.nih.gov/ij/, 1997–2006). The length between each two successive branches of leading hyphae was determined and at least 400 measurements were performed for each colony. The entire data set of each strain was used to calculate the average length between branches. The presented data (and calculated standard error) is based on the averages of at least three independent experiments performed with each strain.

To determine conidiation rates, strains were grown in a 100 ml Erlenmeyer flasks containing 20 ml solid Vogel's sucrose minimal medium. The flasks were incubated for several days at 34°C until conidia were produced and matured. Conidia were collected by adding 50 ml of distilled water and vigorously shaking the flasks. The conidial suspensions were filtered through cheesecloth and subsequently centrifuged for 5 min at 3,000 *g* and the supernatant completely removed. The conidia were then re-suspended in 50 ml of distilled water and the number of total CFUs was determined by use of a hemocytometer.

All the results presented are the average of at least three independent experiments, in which minimum of triplicate counts were performed, with 3–5 different siblings of each strain.

### Protein extraction and immunoblotting

Mycelial samples were frozen in liquid nitrogen, pulverized, and suspended in lysis buffer (1 M sorbitol, 10 mM HEPES [pH 7.5], 5 mM EDTA, 5 mM EGTA, 5 mM NaF, 0.1 M KCl, 0.2% Triton X-100, complete protease inhibitor mixture [Roche Applied Science, Mannheim, Germany). The samples were homogenized by 10 strokes of pestle A in a Dounce homogenizer. The homogenates were centrifuged for 40 min at 10,000 *g*, and the supernatants were recovered and stored at –70°C until analyzed. Proteins were separated by NuPAGE® Bis-Tris 4–12% SDS-PAGE (Invitrogen, Carlsbad, Ca) or by SDS-PAGE 7.5% gels. Western blotting was performed by standard procedures [23]. Antibodies used throughout this study included α-cMYC (Santa Cruz, CA), SYM24 and SYM10 (EMD Millipore Corporation, Billerica, MA, USA), β-Tubulin (Abcam, Cambridge, United Kingdom), α -COT1 [14] and α-Phosph-Ser CSK the *S. cereviciae* homolog of COT1 [24]. The secondary antibodies used were either goat anti-rabbit IgG-HRP (Santa Cruz, CA) for SYM24, SYM24, β-Tubulin and α-Phosph-Ser CSK or goat anti-mouse IgG-HRP (Santa Cruz, CA) for α-cMYC.

Immunoprecipitation of the tagged proteins by either α-cMYC or α-COT1 antibodies was performed according to Maerz *el al*. [25]. For protein detection in polyacrylamide gels the Pierce Silver Stain Kit (Rockford, IL) was used, according to the manufacturer's protocol.

### Microscopy

Light and fluorescent microscopy was performed with a Zeiss Axioscope microscope equipped with a Nikon DXM1200F digital camera.

## Results

### Identifying PRMT-encoding genes and constructing deletion mutants

The genome of *N. crassa* contains three PRMT genes, which have typical arginine methyltransferase domains. NCU07459 (designated *amt-1*), is homologous to the human PRMT1, NCU01669 (*amt-3*) is homologous to PRMT3, and NCU01613 (*skb-1*) is homologous to PRMT5 [and the *S. cerevisiae*, as well as *S. pombe* Shk1-binding protein (Skb1)]. While knockout strains of *amt-1* and *amt-3* (the only two predicted type I arginine methyltransferase genes in *N. crassa*), were available, we produced an *skb-1* (a predicted type II arginine methyltransferase) mutant as a part of this study. To do so, we used a gene replacement cassette (obtained from the FGSC), and transformed the *N. crassa* Δ*mus-52* strain. Hygromycin resistant transformants were isolated and disruption of the *skb-1* gene was confirmed by PCR and Southern blot analysis. All the PRMT-encoding deletion strains produced in this study were viable, although radial growth was slower than the wild type (see below).The *amt-1*;*skb-1* double mutant, also exhibited abnormal hyphal morphology (Fig S1).

**Figure 1:**
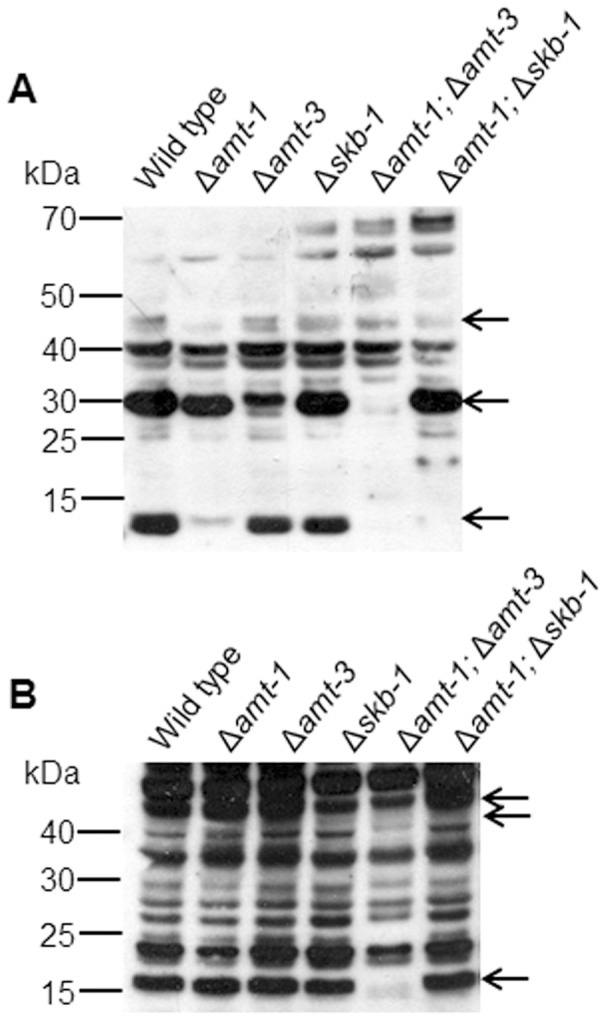
Changes in arginine methylation profiles in PRMT-encoding gene deletion strains. Western-blot analysis of total cell extracts probed with: (A) SYM24 antibodies used to identify asymmetric dimethylarginine and (B) SYM10 antibodies used to identify symmetric dimethylarginine. The most prominent proteins in which differences in the Arg-methylated signals were detected are marked by arrows.

In order to determine whether inactivation of the PRMT-encoding genes conferred changes in arginine methylation, we performed western blot analyses, with specific anti-methyl arginine antibodies, on protein extracts obtained from the mutants (Fig. 1). Antibodies used were either SYM24, that identify asymmetric dimethylarginine, or SYM10 that identify symmetric dimethylarginine. Use of these different antibodies allowed us to distinguish between the outcomes of inactivation of the different PRMT-encoding genes. Differences in the asymmetric dimethylarginine protein profile were observed in Δ*amt-1* and Δ*amt-3* (the predicted type I PRMTs), while no such changes were observed in the Δ*skb-1* strain (Fig. 1A). At the same time, minor differences in symmetric dimethylarginine were observed in Δ*skb-1*, but not in the other single deletion strains (Fig. 1B).

In order to further examine the involvement of the PRMTs in the arginine methylation pattern and to identify possible overlapping functions between the different PRMTs, we crossed deletion strains to produce the following double mutants: Δ*amt-1*;Δ*amt-3* (in which the two identified Type I PRMT-encoding genes were inactivated) and Δ*amt-1*;Δ*skb-1* (in which a single Type I and a Type II PRMT-encoding gene were inactivated). The methylation profile of the Δ*amt-1*; Δ*amt-3* mutant detected by Western blot analysis indicated that deletion of these two putative Type I PRMTs affected not only the asymmetric arginine dimethylation, but also the symmetric arginine methylation profile. Furthermore, the Δ*amt-1*;Δ*skb-1* asymmetric dimethylarginine profile was similar to that observed in the *amt-1* deletion strain, and not that observed in Δ*skb-1* protein extracts. Based on the methylation profiles of the single and double deletion mutants tested, we concluded that arginine methylation was affected in all mutant combinations and that all three genes most likely encode PRMTs. Moreover, the results obtained in the western blot analyses suggest the presence of some reciprocal interactions between different PRMTs.

### Deletion of PRMT-encoding genes affects fungal morphology and physiology

In order to further understand the role of the PRMT-encoding genes in the development of *N. crassa*, we monitored hyphal growth and integrity as well as asexual and sexual spore formation in the different mutants.

**Figure 2:**
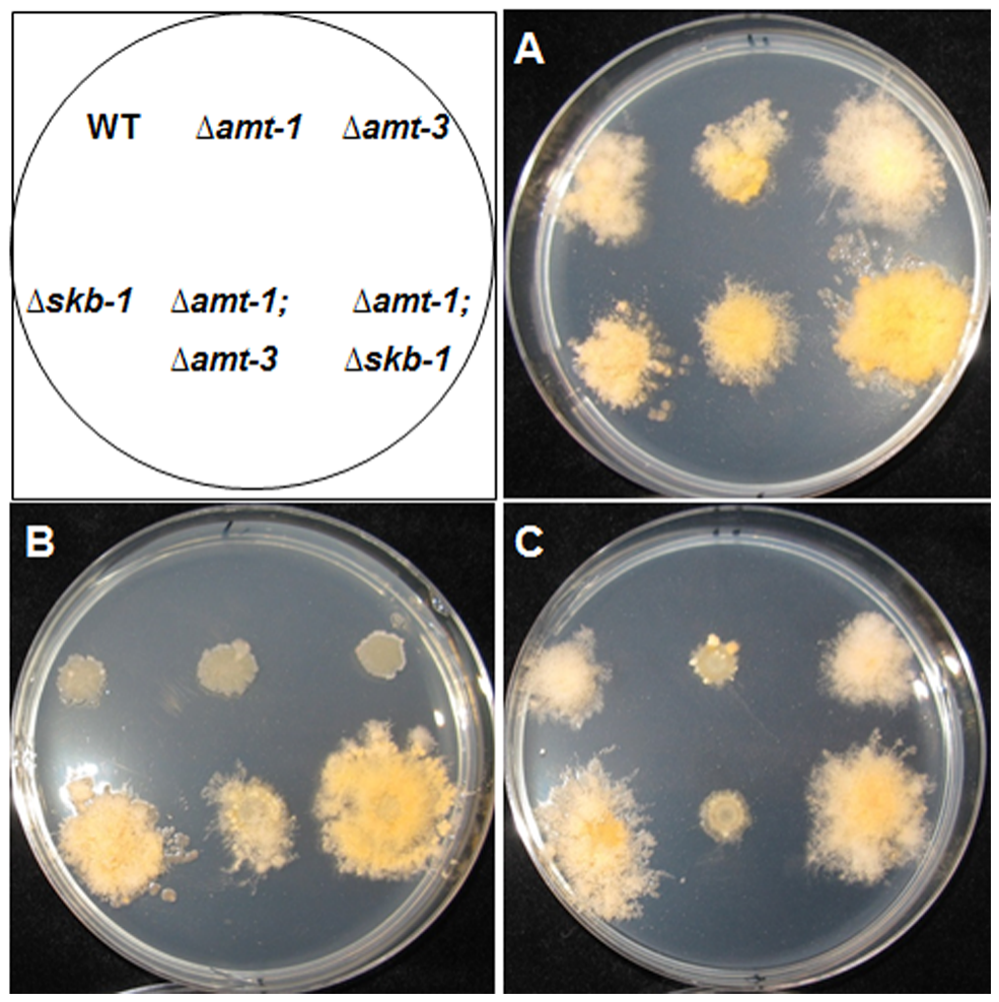
Effect of Polyoxin D and Voriconazole on growth of *N. crassa* PRMT mutants. All strains were cultured on sorbose (2%)-amended (which restricts colony growth) medium at 34°C. (A) control (B) with PolyoxinD (6.5 mM) (C) Voriconazole (12.23 mM).

All the PRMT deletion mutants exhibited significantly reduced hyphal elongation rates, though to different extents (Table 2). Δ*amt-1* grew at a rate that was approximately 30% slower than the wild type, while Δ*skb-1* and Δ*amt-3* exhibited a milder, albeit still significant, reduction (∼16% and 8%, respectively) in hyphal elongation rates. Δ*amt-3* was also affected in its branching frequency, as distance between branches increased by 15%. Amongst the different types of asexual conidia produced by *N. crassa*, under standard growth conditions, macroconida are the most abundant (over 90% of CFUs; [26]). Deletion of *skb-1* had a very marked effect on conidiation, almost doubling the number of macro-conidia produced, while ▵*amt-1* and ▵*amt-3* had no observable effects on this developmental process. We examined conidial viability and germination rates in all the PRMT mutants and found no significant effect of the deletion on these parameters. Furthermore, all three mutants did not exhibit defects in sexual reproduction (as has been observed in other fungi; [7], [9]). To further determine the possible effects conferred by deletion of the PRMT-encoding genes on the hyphal cell, we examined colony growth rates upon exposing the mutants to a variety of stresses known to affect cell integrity (Fig 2). We found Δ*skb-1* and Δ*skb-1;*Δ*amt-1* to be less sensitive to the chitin synthase inhibitor Polyoxin D (Fig. 2B), and no inhibition was detected in these mutants at the EC_25_ wild type level tested. In contrast, no change in sensitivity was observed when the mutants were cultured in the presence of the glucan synthesis inhibitor Caspofungin (data not shown), suggesting that this PRMT may be involved in regulation of cell wall biosynthetic machinery, specifically chitin synthases. The hypersensitivity of *amt-1* (but not Δ*amt-3* or Δ*skb-1*) to Voriconazole (Fig. 2C) suggests ergosterol synthesis or membrane maintenance may also be affected following the impairment of PRMTs.

**Table 2:** Morphological consequences of deletion in PRMT-encoding genes.

Developmental process	Wild type	Δ*amt-1*	Δ*amt-3*	Δ*skb-1*	Δ*amt-1*; Δ*amt-3*	Δ*amt-1*; Δ*skb-1*
Hyphal growth rate (mm/h)	5.3±0.07^A^	3.54±0.18^DE^	4.88 ±0.04^B^	4.47 ±0.13^C^	3.89±0.19^D^	3.26 ±0.07^E^
Length between branches (µm)	162±5^B^	154±4^B^	183±5^A^	159±4^B^	154±3^B^	174±4^A^
Total CFU (*10^6^)	72±4^B^	68±9^B^	79±9^B^	134±7^A^	73±4^B^	127±8^A^

Growth, branching and conidiation of single and double deletion of PRMT-encoding genes. Growth rate (mm/h), length between branches (µm), conidiation, (total CFU *10?6). All experiments were conducted at 34°C, in triplicates. Different letters were used to mark statistically significant difference (P = 0.05).

### SKB1 physically interacts with COT1, and affects its protein abundance

**Figure 3:**
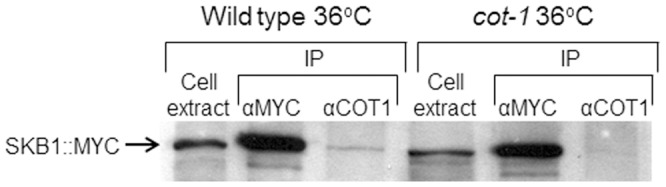
Co- Immunoprecipitation of MYC-SKB1 and COT1. Corresponding proteins were detected with anti-Myc, in wild-type and *cot-1* ts backgrounds. The experiment was performed under restrictive temperature (36°C). “|Cell extract” represents control without immunoprecipitation.

In *N. crassa,* the NDR kinase-encoding gene *cot-1* was previously described to be involved in hyphal elongation and branching, condiation and cell wall integrity [15], [16], [27], all of which seem to be affected, at various levels, by deletion of the PRMT-encoding genes studied here, suggesting a possible functional link between PRMTs and COT1. Furthermore, the presence of a physical interaction between Skb1p and Orb6p (a COT1 homologue) were reported in *S. pombe* [18]. Thus, in order to determine if SKB1 and COT1 physically interact in *N. crassa*, we generated an SKB1::MYC fusion construct, under the control of the native *skb-1* promoter (pME10) and inserted it into the wild-type and *skb-1* strains. The SKB1::MYC construct successfully complemented the ▵*skb-1* mutant and normal conidiation levels and growth rate were restored. A co-immuneprecipitation procedure was conducted using both anti-Myc and anti-COT1 antibodies, and the resolved proteins were subjected to western blot analysis using anti-Myc antibodies (Fig 3, left panel). The results obtained confirm the existence of a physical interaction, *in vivo*, between SKB1 and COT1. The specificity of this interaction is substantiated by the significantly lower abundance of SKB1 in a *cot-1* ts background (Fig. 3, right panel), in which COT1 is less stable [27]. To further explore whether this physical interaction may affect COT1 intercellular localization and protein levels, we crossed the *skb-1* deletion mutant with MYC::COT1, and COT::GFP strains. No changes were observed in COT1::GFP localization in the Δ*skb-1* background, and COT1 was clearly observed at the hyphal apical region (Fig. S2), as has been previously described [28].

**Figure 4:**
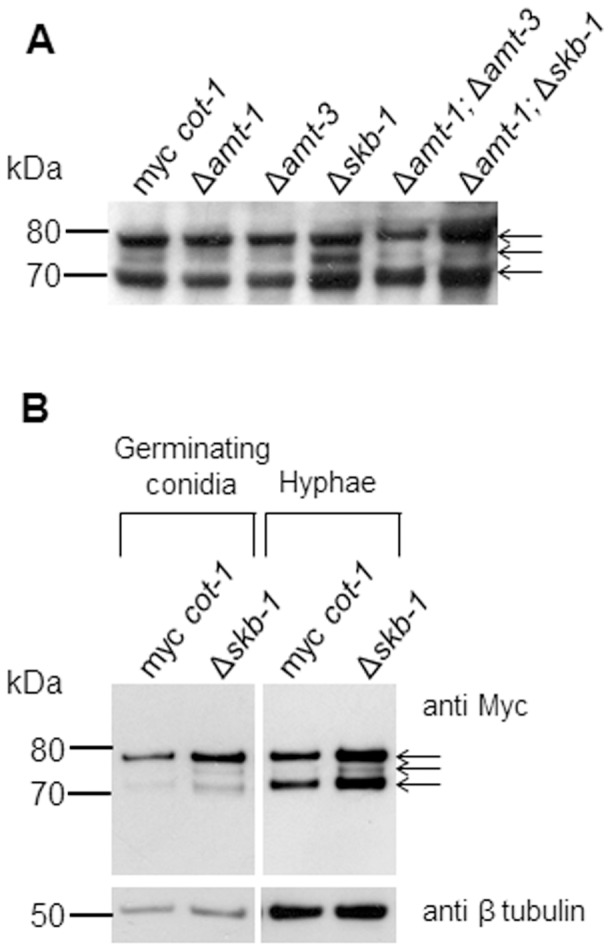
Deletion of *skb-1* affects COT1 protein level. Western-blot analysis of total cell extracts probed with anti-Myc antibodies (A) Single and double PRMT deletion strains in a MYC::COT1 background (B) MYC::COT1 in aΔ*skb-1* background at two developmental stages. β tubulin levels are presented as controls for each developmental stage examined.. MYC::COT1 isoforms are marked by arrows.

However, the deletion of *skb-1* did affect the abundance of COT1 protein levels. Recently, 3 isoforms of COT1 (tagged with a 6xMYC tag) have been identified and characterized, referring to protein weights of 70, 75 and 80 kDa [29]. The 70 and 80 kDa proteins correspond to the two previously described COT1 isoforms [14]. In the Δ*skb-1* background, the abundance of the mid-size isoform (75 kDa), was significantly elevated (Fig. 4A). We explored this further, and looked at COT1 protein levels during different developmental stages, and found that the absence of *skb-1* results in altered COT1 protein levels as early as during conidial germination (Fig. 4B).

**Figure 5:**
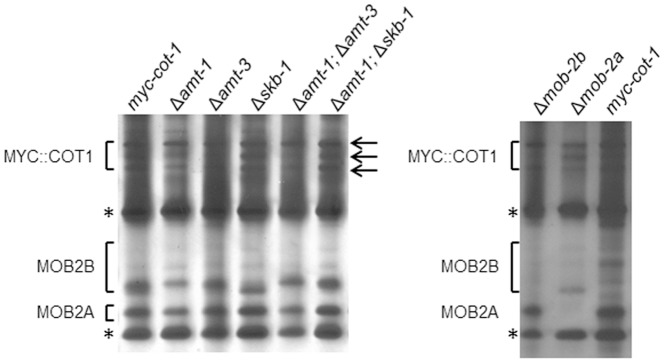
Deletion of *skb-1* does not affect COT1 interaction with MOB2 co-activators. Immunoprecipitation of MYC::COT1 with anti-Myc antibodies. Proteins obtained were resolved on a gradient gel and subjected to silver staining. MOB2A and MOB2B co-precipitants are marked. MYC::COT1 isoforms are marked by arrows. * marks the IgG.

COT1 has been shown to interact with co-activators from the MOB family, MOB2A and MOB2 [25]. In order to determine whether this interaction is dependent on the physical interaction between COT1 and SKB1, we preformed co-immunopercipitation experiments with proteins in a Δ*skb-1* background, and found that the interaction between COT1 and MOB2A or MOB2B (Fig. 5) was not altered. These results may indicate that SKB1 is not required for COT-MOB2A/B binding, yet may be a part of the COT1-MOB2 complex.

### COT1 is methylated on Arginine residues

**Figure 6:**
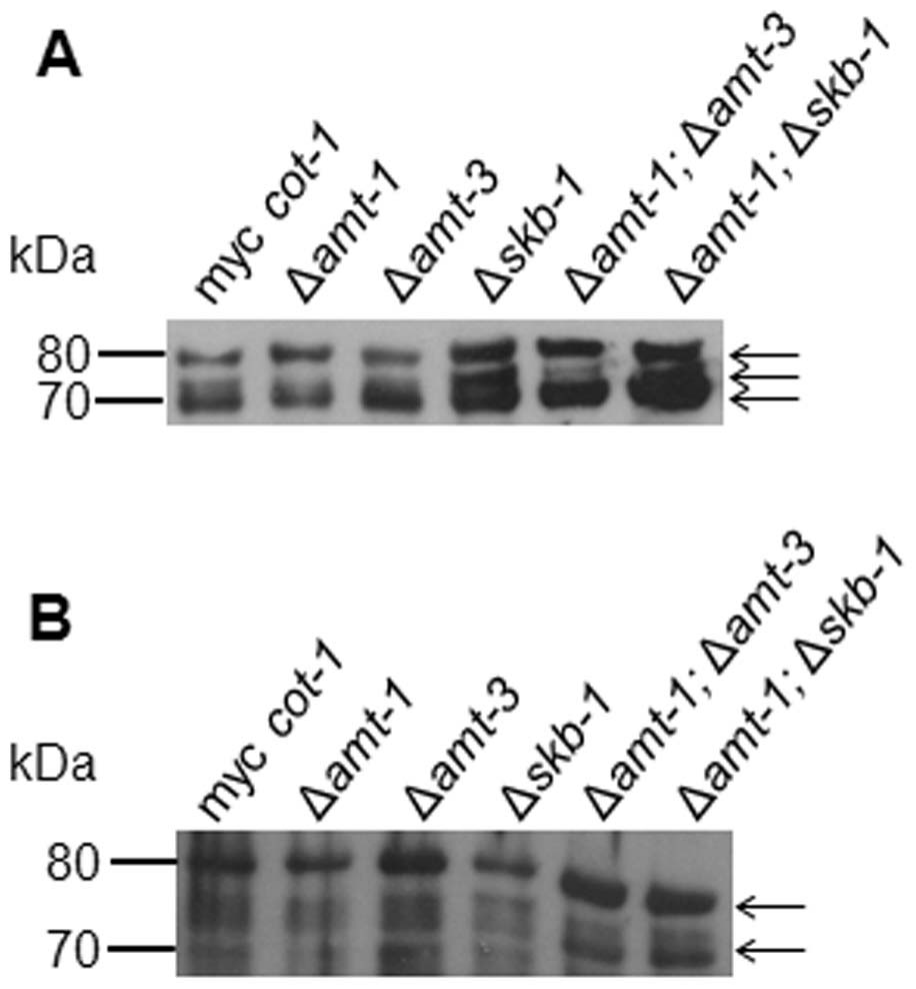
Arginine methylation on MYC::COT1. Immunoprecipitation of MYC::COT1with anti-Myc antibodies probed with: (A) SYM24 antibodies (detecting asymmetric dimethylarginine) and (B) SYM10 antibodies (detecting symmetric dimethylarginine). MYC::COT1 isoforms are marked by arrows.

Based on the detection of a physical interaction between COT1 and SKB1, we examined whether COT1 undergoes arginine methylation. COT1 proteins were immunopercipited from MYC::COT1 strain extracts and the 3 isoforms (80, 75 and 70 kDa) probed for arginine methylation using the antibodies described above. When the SYM24 antibodies (specific for asymmetric dimethylarginine residues) were used, all three isoforms were detected (Fig. 6A). The SYM10 antibodies reacted only with the 80 kDa isoform (Fig. 6B). These analyses demonstrate that all COT1 isoforms are methylated on Arginine residues, though the nature of the methylation differs amongst them. We used the same approach (using the *myc-cot-1* strain) to determine whether the Arg methylation state on the different COT1 isoforms is dependent on SKB1 or the other PRMT-encoding genes. Unexpectedly, no observable changes in COT1 arginine methylation signals were detected in extracts obtained from single PRMT-encoding gene mutants (taking into consideration that an increase in COT1 abundance was detected in a Δ*skb-1* background, as shown in Fig 4). However, in the Δ*amt-1*;Δ*amt-3* and Δ*amt-1*;Δ*skb-1*, the SYM10 antibodies could detect both the 80 and 70 kDa isoforms of COT1 (Fig. 6B). As COT1 was detected with both asymmetric and symmetric dimethylarginine antibodies, we hypothesize that it is likely that more than one PRMT is involved in the process.

In the light of these results we explored the possibility that other PRMTs, besides SKB1, may be involved in COT1 regulation. It seems that COT1 protein abundance is elevated in the Δ*amt-1;*Δ*skb-1* mutant, but no apparent changes occurred in the other deletion strains (Fig. 4A). Furthermore, utilizing specific antibodies, we have not found significant differences in the ability of Ser417, which is the COT1 autophosphorylation site [15], [28], to undergo phosphorylation (Fig. S3). In addition, no changes were found in the ability of COT1 to interact with the MOB2 co-activators (Fig. 5). Taken together, it appears that if Arg methylation in COT1 is dependent on PRMTs, COT1 autophosphorylation and MOB2A/B protein binding are not affected by the lack of these enzymes.

### PRMTs genetically interact with COT1

**Figure 7:**
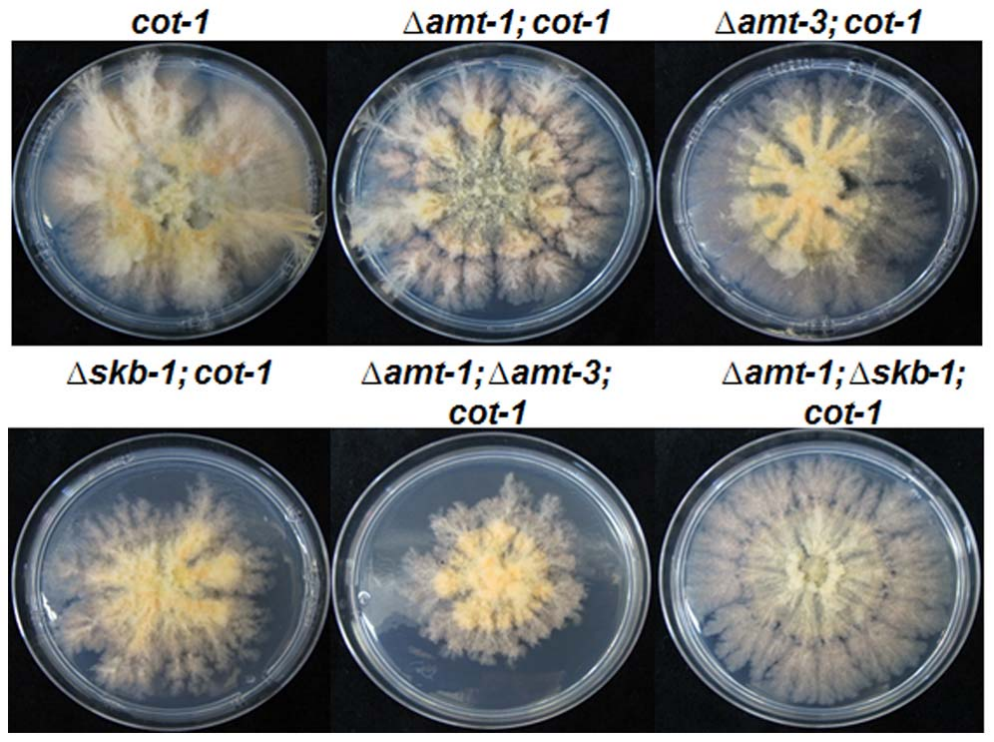
Colony morphology of PRMT- encoding gene deletion strains in a *cot-1* ts background. Strains were cultured for 72 hours at 30°C (a semi-restrictive temperature).

Since we suspect that at least two PRMT are involved in COT1 arginine methylation, we examined if *skb-1* or *amt-1*/*amt-3* can genetically interact with *cot-1*. To do so we used the *cot-1* ts mutant, in which changes are conferred in COT1 isoform abundance when the strain is grown at a temperature above 32°C, conditions in which growth arrest and hyper branching occur [14], [16]. We crossed the single or double PRMT-encoding gene deletion strains into the *cot-1* ts background, and examined the morphology and growth rate at a semi-restrictive temperature of 30°C. The morphology of the all single and double PRMT-encoding gene deletion strains in a *cot-1* ts background was different from that observed in *cot-1* ts (Fig. 7). This was mainly characterized by a significant increase in growth rates of all the strains at a semi-restrictive temperature (in comparison to permissive conditions; Table 3). At permissive temperatures no differences were observed in relation to the parental strains in a non-*cot-1* ts background (data not shown).

**Table 3:** Growth rate of PRMT-encoding genes deletion strains in a *cot-1* ts background.

Radial growth rate	Δ*amt-1*	Δ*amt-3*	Δ*skb-1*	Δ*amt-1*; Δ*amt-3*	Δ*amt-1*; Δ*skb-1*
25°C (% from *cot-1*)	77.6%±3.1%	95.4%±2.5%	86.4%±0.8%	64.8%±0.1%	64.2%±0.4%
30°C (% from *cot-1*)	89.4%±2.1%	128%±4%	105.8%±3.3%	88.6%±4.9%	83.9%±2.2%

Growth rate is presented as % of *cot-1* ts. Experiments were conducted at 30°C or 25°C (semi-restrictive or permissive temperatures, respectively), in triplicates.

**Figure 8:**
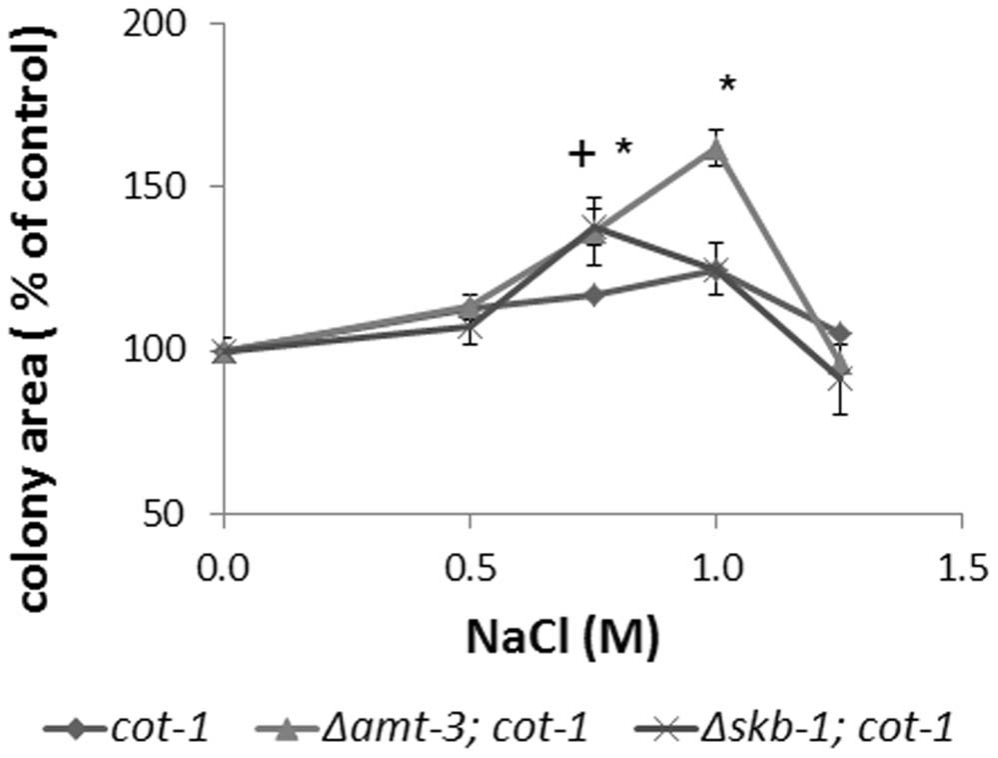
Effect of inactivation of Δ*amt-3* or *Δskb-*1 on growth of *cot-1* ts. *cot-1* ts, grown in the presence of NaCl, exhibits a suppressed colonial phenotype. The colonial phenotype is further suppressed upon inactivation of *amt-3* or *skb-1*. Strains were cultured for 72 hours at 34°C (*cot-1* ts restrictive temperature) with 0.5, 0.75, 1 or 1.25 M NaCl. Colony area is presented as percent of control. * and + indicate a significant difference (p<0.05) of colony area in the Δ*amt-3*;*cot-1* or Δ*skb-1*;*cot-1* backgrounds, respectively.

The *cot-1* mutation is environmentally suppressed at restrictive temperature by osmotic or oxidative stresses [27]. We examined the ability of the various Δ*amt-1/3;cot-1* and Δ*skb-1*;*cot-1* strains to undergo phenotypic suppression by NaCl. In both Δ*skb-1* and Δ*amt-3* backgrounds, the degree of phenotypic suppression of *cot-1* was significantly increased, suggesting that both these PRMT-encoding genes may be involved in environmental sensing by COT1 (Fig. 8).

## Discussion

### Different PRMT deletion mutants exhibit variations in Arg methylation patterns

Eukaryotic cells extend their polypeptide diversity beyond constrains of the encoded amino acid sequence and folding by means of post translational modifications. The understanding of the significance of these changes for fungal growth and development processes has risen in the last years [30]. One such modification is Arg methylation, carried out by protein arginine methyltransferases. This family of enzymes is conserved from yeast to human, and regulates stability, localization and activity of proteins [2]. Systematic analysis of the involvement of the PRMT family in fungal morphology was previously explored in *A. nidulans* [7] and *F. graminearum* [9]. Here, we analyzed the three PRMTs of *N. crassa*, with emphasis on the possible involvement of PRMTs in the characteristic rapid hyphal growth of this fungus [31].

The three *N. crassa* genes that contain the arginine methyltransferase domain were identified as: NCU07459 (*amt-1*), NCU01669 (*amt-3*), which comprise the two predicted type I PRMTs, and NCU01613 (*skb-1*), the predicted type II PRMT. Their phylogenetic associations have been previously described [9]. In addition to the three highly conserved PRMTs, another, protein (NCU08111), which may be a putative type IV PRMT, but doesn't contain the conserved methytransferase domain, is present in the *N. crassa* genome. In *F. graminearum* inactivation of the homologue of this gene, which is structurally significantly different than the cross-kingdom classes of PRMTs [5], had no observable phenotypic consequences [9]. The enzymatic characteristics of this enzyme and its involvement in cellular functions in *N. crassa* and additional fungi have yet to be determined.

The amenability of *N. crassa* to genetic manipulations allowed us to construct double deletion mutants of genes from the PRMT gene family: Δ*amt-1*;Δ*amt-3*, that is similar in nature (a double Type I PRMT mutant) to a mutant previously examined in *F. graminearum* [9] and Δ*amt-1*;Δ*skb-1* (combining a Type I and Type II PRMT inactivation) that to the best of our knowledge was produced and examined for the first time during this study.

We used different anti methyl Arg antibodies to explore the profile of proteins that are methylated on arginine residues in PRMT deletion mutants. Our results verified the involvement of the predicted genes in this process. Based on these analyses we concluded that even though both AMT1 and AMT3 perform aDMA, the significant differences in the aDMA profiles observed between the two mutants lacking these enzymes suggest that the their function involves different substrates in *N. crassa*. We have also demonstrated that, as expected, SKB1 preforms sDMA. Examining the Arg methylation protein profiles of Δ*amt-1*;Δ*amt-3* revealed, to our surprise, a significant reduction not only in aDMA, but also in sDMA. Since the Arg methylation profile of deletion mutant in two type I PRMTs has never been examined in other organisms, it has yet to be determined if the results obtained are unique to *N. crassa*. One possible explanation for the observed changes is the presence of reciprocal regulation of the different PRMTs. Specifically, that *amt-1* and *amt-3* are involved in regulation of *skb-1*, and vice versa. Such a form of regulation could function at the protein level, in a manner resembling the way human PRMT 1,4,6,8 can undergo self Arg-methylation [32], or at the transcriptional level, as seen in *F. graminearum* where a 44% reduction in AMT4 (the *skb-1* homologue) transcript abundance was detected in a Δ*amt1* strain [9].

Another interesting finding was in the Arg methylation profiles of the Δ*amt-1*;Δ*skb-1* strain. While in the Δ*skb-1* mutant differences in sDMA were observed, in Δ*amt-1*;Δ*skb-1* no such differences were detected. This further supports our suggestion concerning cooperative/reciprocal regulation of enzymes of the PRMT family.

### Different PRMT deletion mutants affect fungal morphology

In order to improve our understanding concerning the involvement of PRMTs in *N. crassa* morphology and physiology, we analyzed different parameters of fungal growth and development. All strains with single or double deletion of genes encoding for PRMTs exhibited reduced growth rate. This was most pronounced in the Δ*amt-1* strain 30%∼) reduction), and resembles that observed in *F. graminearum* [9], but not in *A. nidulans* [7]. A lesser, yet significant, reduction in growth rate was also observed in Δ*amt-3* and Δ*skb-1*, that so far has not been reported in filamentous fungi. The double deletion mutants exhibited growth rates that were similar to Δ*amt-1*. This is in agreement with that observed in the double deletion strains of *F. graminearum* [9]. These results raise the possibility that all the PRMTs regulate growth through similar cellular pathways, and Δ*amt-1* serves as a rate limiting factor in this regulatory process.

The growth and branching of *N. crassa* hyphae are important for utilization of organic substrates [33]. We identified a new role of Δ*amt-3*, showing it is involved in regulation of hyphal branching, as inactivation of this gene significantly increased (∼15%) the length between branches, reducing the number of branches in the hyphal colony. This, along with the fact that information on function of PRMTs in fungi, as well as in other organisms is lacking [5] (, warrants further investigation on the roles AMT3 plays.

The *skb-1* gene was the only PRMT-encoding gene that was clearly involved in asexual sporulation, as its deletion conferred a doubling in the number of macro-conidia, that represent 90% of all a-sexual conidia in *N. crassa* [26]. However, no such involvement has been reported in other conidia-producing fungi [7], [9]. Moreover, observations described here suggest that *skb-1* may be involved in chitin synthesis, an important component of the fungal cell wall. The hypersensitivity of Δ*amt-1* to Voriconazole suggests it may be involved in ergosterol synthesis or other aspects of membrane integrity. This is in line with the growth reduction in the presence of SDS which was previously described in *F. graminearum,* where *AMT1* was suggested to be involved in responses to membrane stresses [9]. Interestingly, although deletion mutants of PRMT1 in other fungi exhibited sensitivity to oxidative stress, we have not observed a similar phenotype in *N. crassa* (data not shown).

### COT1 is methylated on Arg residues

The PRMTs in *N. crassa* affect morphological aspects of fungal growth and development (e.g., hyphal elongation, branching, condiation, and chitin synthesis), which were previously reported to be regulated by COT1 [15], [16], [27]. COT1 is an NDR kinase, which is a subclass of the AGC class of Ser/Thr protein kinases [13]. Members of the NDR family, as are those of the PRMT family, are highly conserved, from fungi to mammals. Using antibodies that identify methylation on Arg residues, we found the presence of these modifications on COT1. While all three COT1 isoforms can undergo aDMA, only the 80 kDa isoform exhibited sDMA. The most significant change in arginine methylation was detected in the double PRMTs deletion strains, so it seems that COT1 is modified by more than one enzyme from this family. This does not rule out minor changes in methylation which may occur in the single mutants but are difficult to quantify on the basis of the analysis performed here.

### SKB1 physically interacts with COT1, and affects its protein abundance

Previous work in *S. pombe* identified a physical interaction between SKB1 and Orb6p (the COT1 homologue) [18]. We established that a similar interaction occurs in *N. crassa*, verifying its existence by co-immunoprecipitation. We also determined that deletion of *skb-1* did not affect the interaction between COT1 and its co-activators (MOB2A/B), suggesting that the interaction between these activators and COT1 is not dependent on the presence of SKB1. In order to better understand the nature of PRMT involvement in COT1 function, we examined the influence of PRMT-encoding gene inactivation on COT1 protein levels and found an increase in the abundance of the COT1 75 kDa isoform in Δ*skb-1*. Interestingly, this was less pronounced in the Δ*amt-1*;Δ*skb-1* strain, though a clear signal was still observed. As some of the features of this double mutant were more similar to the Δ*amt-1* strain, it is possible that *amt-1* has a more marked effect on the abundance of the 75 kDa isoform, even in a *skb-1* background. The 75 kDa COT1 isoform is suggested to be the result of a yet to be identified post-translational modification of COT1 and its abundance is reduced following changes in the N terminal region (NTR) of COT1 [29]. It is tempting to speculate that changes in the NTR (e.g., extent of Arg methylation or via interaction with other proteins), as conferred by SKB1, may be involved in the changes observed in abundance of the 75 kDa isoform.

### PRMT-encoding genes genetically interact with *cot-1*

We examined PRMTs ability to genetically interact with COT1, using a *cot-1* temperature sensitive mutant, which exhibits restricted, colonial growth under restrictive temperatures. The results obtained suggest that all deletions in PRMT encoding genes affect the morphology and growth rate of a *cot-1* ts mutant. Examination of the ability of the deletion strains to undergo environmental suppression at restrictive temperature, which is typical to the *cot-1* ts mutant [14], showed that growth rate of *cot-1* was increased in both Δ*amt-3* and Δ*skb-1* backgrounds, when grown in the presence of high salt concentrations. Thus, these PRMTs may be linked with environmental sensing functions associated with COT1.

## Conclusions

In this study we have progressed our understanding of the PRMT family in regulation of morphology and physiology in *N. crassa*, and have identified previously unknown roles for these genes/enzymes. Construction of double deletion PRMT –encoding gene mutants suggest there is reciprocal, direct or indirect, regulation of enzymes in this family. From the double mutants analysis it seems that there is an epistatic relationship in different cellular processes; the deletion of *amt-1* was epistatic to *amt-3* in all morphological characteristics. We have shown, for the first time, that the NDR kinase COT1undergoes Arg methylation and genetically interacts with PRMTs. These data demonstrate the importance of methylation as a modification involved in regulation of a protein kinase. Furthermore, the increased phenotypic suppression of the *cot-1* ts phenotype under osmotic stress conditions indicates the involvement (direct or indirect) of PRMTs in COT1-associated environmental sensing. Based on the highly conserved structure of the PRMTs and the NDR kinases in all eukaryotic systems, we propose that these proteins have similar regulation and modes of action, and therefore our findings may be relevant to other fungi and higher eukaryotes.

## Supporting Information

Figure S1 **Fungal morphology of the PRMT-encoding gene deletion strains.** Hyphal morphology at the edge of the colony of wild type and PRMT-encoding gene deletion strains cultured overnight at 34°C. Arrow marks abnormal hyphal growth. Bar  =  100 µm.
(TIF)

Figure S2 **Deletion of** ***skb-1*** **does not affect COT1::GFP hyphal tip localization.** Localization of COT1::GFP in wild type (A) and in a Δ*skb-1* (B) backgrounds. Strains were cultured overnight at 34°C.
(TIF)

Figure S3 **Deletion of PRMT-encoding genes does not impair Ser417 phosphorylationin COT1.** Anti-Ph-Ser-CBK1 antibodies were used to detect the phosphorylated COT1 isoforms, following immunoprecipitation, with anti-Myc antibodies in various PRMT-encoding gene mutant backgrounds. MYC::COT1 isoforms are marked by arrows.
(TIF)
