# Supplementary figures and images for: Neurospora crassa Protein Arginine Methyl Transferases Are Involved in Growth and Development and Interact with the NDR Kinase COT1
Source: PLoS One. 2013 Nov 19;8(11):e80756. doi: 10.1371/journal.pone.0080756 (PMC3834314; doi:10.1371/journal.pone.0080756)

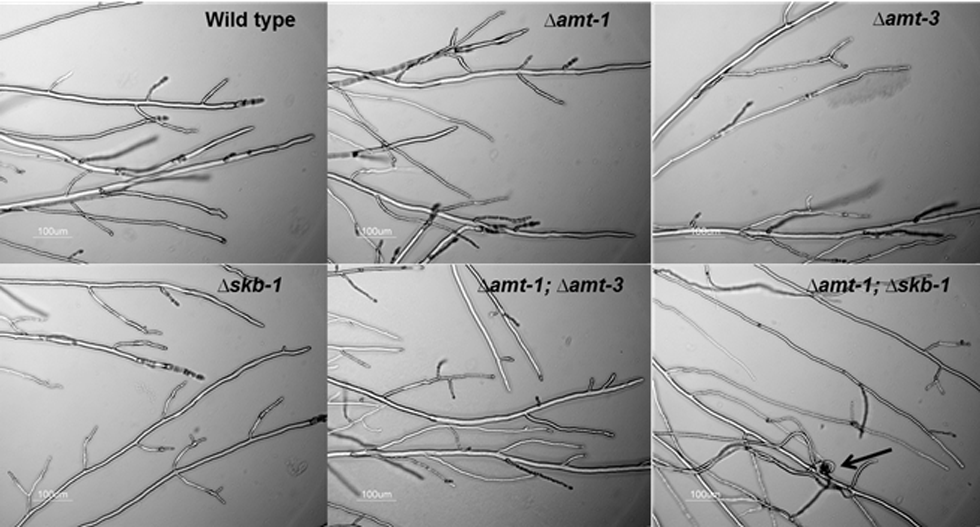

Supplement: Figure S1 — Fungal morphology of the PRMT-encoding gene deletion strains. Hyphal morphology at the edge of the colony of wild type and PRMT-encoding gene deletion strains cultured overnight at 34°C. Arrow marks abnormal hyphal growth. Bar = 100 µm. (TIF) [file pone.0080756.s001.tif]

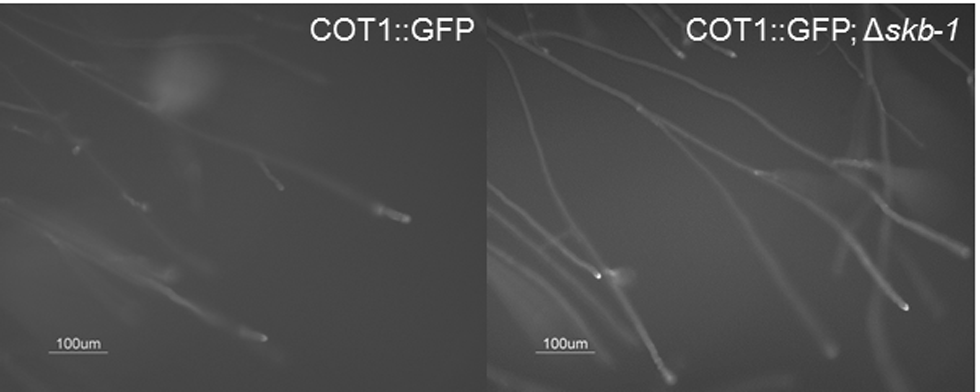

Supplement: Figure S2 — Deletion of skb-1 does not affect COT1::GFP hyphal tip localization. Localization of COT1::GFP in wild type (A) and in a Δskb-1 (B) backgrounds. Strains were cultured overnight at 34°C. (TIF) [file pone.0080756.s002.tif]

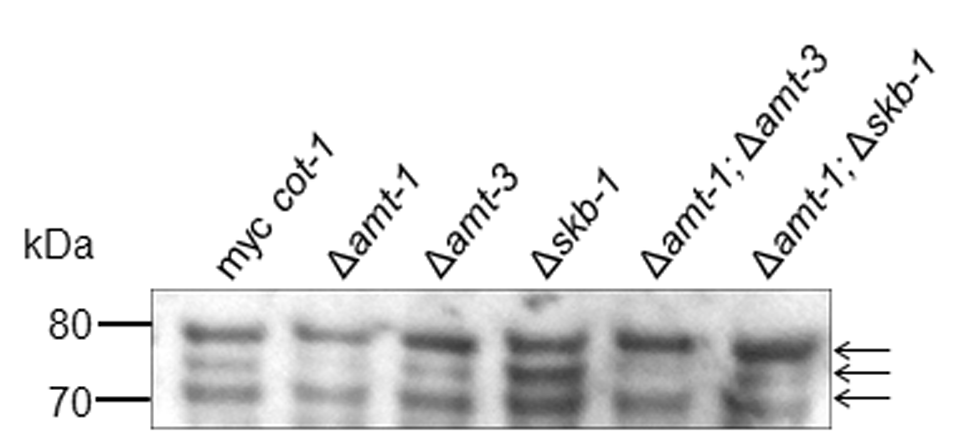

Supplement: Figure S3 — Deletion of PRMT-encoding genes does not impair Ser417 phosphorylationin COT1. Anti-Ph-Ser-CBK1 antibodies were used to detect the phosphorylated COT1 isoforms, following immunoprecipitation, with anti-Myc antibodies in various PRMT-encoding gene mutant backgrounds. MYC::COT1 isoforms are marked by arrows. (TIF) [file pone.0080756.s003.tif]
